# Cartilage-Specific ^18^F-NaF Uptake in Rat Models: A Multimodal In Vitro and Ex Vitro Comparative Study with ^99m^Tc-MDP

**DOI:** 10.3390/biomedicines13071540

**Published:** 2025-06-24

**Authors:** Qingxiao Li, Jianpeng Gao, Yiqun Wang, Yaoyao Song, Liwei Liu, Cong Zhang, Ming Li, Haodan Dang, Jiahe Tian

**Affiliations:** 1 Department of Nuclear Medicine, The First Medical Centre, Chinese PLA General Hospital, Beijing 100853, China; lqx811@163.com (Q.L.); 15314282327@163.com (C.Z.);; 2 Medical School of Chinese PLA, Beijing 100853, China; liuliwei304@126.com; 3 Department of Orthopaedics, Chinese PLA General Hospital, Beijing 100039, China; bonegaojp@163.com; 4 Orthopaedic Sports Medicine Center, Beijing Tsinghua Changgung Hospital, Affiliated Hospital of Tsinghua University, Beijing 102218, China; www9299sss@163.com; 5 Department of Sports Medicine, Peking University Third Hospital, Institute of Sports Medicine of Peking University, Beijing 100080, China; 6 Senior Department of Burns and Plastic Surgery, Fourth Medical Center of Chinese PLA General Hospital, Beijing 100038, China; yaoyao_430@126.com

**Keywords:** ^18^F-NaF, ^99m^Tc-MDP, bone imaging, calcification, multimodal imaging, early osteoarthritis

## Abstract

**Background:** ^18^F-NaF and ^99m^Tc-MDP are widely used bone imaging tracers, but their comparative uptake in bone versus cartilage is unclear. This study aimed to directly compare these patterns in rats to guide musculoskeletal molecular imaging. **Methods:** Male Sprague-Dawley rats underwent in vivo and ex vivo radiotracer studies. Tracer uptake (%ID/g) was quantified in bone and cartilage at 30, 60, or 120 min post-injection (^18^F-NaF or ^99m^Tc-MDP), and across different ages. Additional rats received subcutaneous implants of viable or devitalized bone and cartilage; uptake was assessed using PET/CT, autoradiography, and histology. **Results:** ^18^F-NaF showed faster blood/background clearance and higher target-to-background ratios compared to ^99m^Tc-MDP, especially in weight-bearing joint cartilage. ^18^F-NaF uptake in cancellous bone significantly exceeded that of ^99m^Tc-MDP, whereas ^99m^Tc-MDP showed higher uptake in knee cartilage. Age-related analysis showed maximal knee cartilage accumulation in aged rats. Histological and cell inactivation studies confirmed that ^18^F-NaF uptake reflects both cellular activity and degree of calcification. **Conclusions:** ^18^F-NaF demonstrates distinctive, quantifiable uptake in cartilage, dependent on both cellular activity and calcification, and exhibits favorable imaging characteristics versus ^99m^Tc-MDP for cartilage metabolism. These findings support ^18^F-NaF as a promising tool for early diagnosis and therapeutic monitoring of bone and joint disorders, and provide pathophysiological insight into the dynamics of the bone–cartilage interface.

## 1. Introduction

Bone imaging, a pivotal nuclear medicine technique, relies on the specific visualization of osseous metabolic activity through radiopharmaceutical localization to provide critical diagnostic and therapeutic insights. Traditional agents like technetium-99m-methylene diphosphonate (^99m^Tc-MDP) bind to hydroxyapatite crystals, effectively reflecting bone resorption and remodeling processes, and have been widely applied in clinical practice [1]. In recent years, ^18^F-sodium fluoride (^18^F-NaF) has emerged as a superior PET tracer for bone metabolic imaging due to its enhanced bone uptake kinetics, rapid blood clearance, and minimal soft-tissue retention [2]. However, existing research predominantly focuses on osseous metabolism, with insufficient attention to cartilage, a biomechanically and biochemically critical component of the skeletal system [3].

Cartilage serves as a critical mediator in bone growth, repair, and pathological processes, where longitudinal monitoring of its metabolic status provides essential insights into bone–cartilage interface dynamics. However, chondrocyte calcification exhibits fundamentally distinct metabolic pathways compared to bone mineralization [4,5]. Developmental biology studies reveal that cartilage calcification constitutes a dynamic cascade involving calcium salt deposition, angiogenesis, and cellular activity modulation processes indispensable for physiological bone development and pathological remodeling [6,7]. Despite this significance, the uptake characteristics and biological implications of conventional bone-seeking tracers in cartilage remain poorly characterized [8]. Current research predominantly focuses on quantitative bone metabolism analysis [9,10,11], while cartilage is frequently dismissed as background tissue without systematic investigation [12,13,14,15]. Furthermore, conventional SPECT imaging suffers from limited spatial resolution (8–10 mm), impeding precise differentiation of cartilage–bone metabolic activities. In contrast, PET tracers like ^18^F-NaF achieve superior resolution (2 mm) [16], and their integration with multimodal PET/CT or PET/MRI systems significantly enhances sensitivity and accuracy in tracking both chondral and osseous metabolic events [17,18,19,20]. These technological advances underscore the necessity to investigate ^18^F-NaF uptake patterns in cartilage and their correlation with bone metabolism, a critical step toward expanding the diagnostic utility of molecular bone imaging.

This study aims to investigate the molecular imaging features of cartilage metabolic activity by comparing the differential uptake patterns of ^99m^Tc-MDP and ^18^F-NaF in rat cartilage and bone tissues. Through integrated in vivo and in vitro experiments, we seek to validate the cartilage-specific uptake of ^18^F-NaF, elucidate the molecular mechanisms underlying cartilage calcification and its dynamic interplay with bone metabolism, and establish quantitative diagnostic criteria for metabolic activities in both tissues. By addressing these objectives, this work is expected to provide novel tools for early diagnosis and clinical evaluation of bone defect repair and osteoarthritic diseases. Furthermore, it aims to advance the understanding of cartilage metabolism through multimodal imaging approaches, thereby laying a foundation for optimizing therapeutic strategies and improving diagnostic precision in osteoarthritic pathologies.

## 2. Methods

This study was designed and reported in strict compliance with the ARRIVE 2.0 guidelines and underwent thorough review by the Experimental Animal Ethics Committee of the People’s Liberation Army General Hospital (PLAGH) [21]. Ethical approval was also obtained from the Biomedical Ethics Committee of Peking University Third Hospital (Approval No. LA2020353, approved on 19 May 2020). All animal procedures were conducted in accordance with relevant international and institutional guidelines for the care and use of laboratory animals, ensuring animal welfare and ethical standards throughout the study. All experimental procedures were performed under isoflurane anesthesia (2–3% in oxygen-enriched carrier gas) to ensure adherence to animal welfare standards and experimental rigor.

### 2.1. In Vitro Biodistribution Experiment

Tissue samples were washed with saline, dried, and weighed using an electronic balance (accuracy ±0.1 mg). Subsequently, the radioactive activity of the tissues was measured using a γ-counter (PerkinElmer 2480, Shelton, CT, USA). Radioactive uptake values were expressed as %ID/g, calculated using the formula: %ID/g = (tissue activity (kBq)/injected dose (kBq)) × (1/tissue mass (g)) × 100%. All data were time-decay corrected based on the physical half-lives of the radionuclides (^18^F: 109.8 min; ^99m^Tc-MDP: 6.01 h) to ensure accuracy and comparability of results.

### 2.2. Time-Dependent Biodistribution Study

Twenty-four 4-month-old male Sprague-Dawley rats (weight 250 ± 20 g) were randomly assigned to two experimental groups: the ^18^F-NaF group (n = 12) and the ^99m^Tc-MDP group (n = 12). The ^18^F-NaF group received a tail vein injection of 0.74 MBq/150 μL of ^18^F-NaF (specific activity 200–250 GBq/μmol), while the ^99m^Tc-MDP group received an equivalent radioactive dose. Each group was further divided into three subgroups (n = 4/group), with euthanasia performed at 30, 60, and 120 min post-injection. During dissection, tissue samples were collected, including background tissues (heart, liver, spleen, lung, kidney, brain, and quadriceps), bone tissues (femoral cortex, femoral cancellous bone, skull, and patella), and cartilage tissues (weight-bearing cartilage of the femoral head, knee cartilage, meniscus, and xiphoid cartilage).

### 2.3. Age-Related Biodistribution Study

Twelve Sprague-Dawley rats were divided into three age groups: juvenile (4 weeks old, n = 4, weight 50 ± 10 g), adult (4 months old, n = 4, weight 300 ± 20 g), and elderly (14 months old, n = 4, weight 450 ± 30 g). Based on preliminary experimental data, 60 min post-injection was selected as the optimal tracer equilibrium time point. After a tail vein injection of 0.74 MBq/150 μL of ^18^F-NaF, rats were euthanized at the designated time points for sample collection. Bone tissues included the femoral cortex (representing dense bone) and distal femoral cancellous bone (metabolically active region); cartilage tissues included the femoral head (weight-bearing area), knee joint (mechanical load area), and xiphoid process (non-weight-bearing control).

### 2.4. In Vivo Imaging Experiment

#### 2.4.1. Unilateral Implantation Models

Models were developed based on modified literature protocols for hydroxyapatite implantation [22]. Rats were anesthetized with isoflurane gas and received subcutaneous antibiotic injections to prevent infection. The hind leg or dorsal area was prepared by removing fur and disinfecting with iodine solution. A surgical incision was then made either in the left quadriceps muscle or subcutaneously on the back, creating a pocket approximately 3 × 5 × 5 mm in dimension for implantation purposes. This pouch was filled with allogeneic bone and cartilage (bone samples included femoral cortical and cancellous bone, while cartilage samples included the femoral head, knee joint, meniscus, and xiphoid process). All allogeneic bone and cartilage were sourced from other male SD rats of the same litter to minimize individual differences affecting experimental outcomes. The contralateral side underwent a sham surgery, and the pouch and skin were finally sutured closed.

#### 2.4.2. Bilateral Implantation Model

To investigate the uptake mechanisms of the tracer, eighteen SD rats were selected for bilateral implantation of allogeneic bone and cartilage in the quadriceps of both thighs, with the left side designated as the inactive group (frozen inactivated) and the right side as the normal group. The implanted materials included cortical bone, cancellous bone, xiphoid process, meniscus, femoral head cartilage, and knee joint cartilage, with three samples from other male SD rats of the same litter for each group.

#### 2.4.3. PET/CT Imaging Acquisition Method

Experimental animals were placed in an induction chamber for pre-anesthesia with a mixture of 4.0% isoflurane and air. Once inactive, they were secured in a prone position on the scanning bed, with anesthesia maintained on 2.0% isoflurane and air. Following this, ^18^F-NaF was injected via the tail vein, and dynamic Micro-PET/CT scans (Pingseng Kunshan China ) were performed one hour post-injection. The PET data were reconstructed using the 3D ordered subset expectation maximization (3D-OSEM) algorithm. After PET scans, a CT scan was conducted with parameters set to 60 kV tube voltage, 500 μA tube current, and a slice thickness of 1.5 mm, utilizing the Feldkamp–Davis–Kress (FDK) algorithm for reconstruction. All image data were processed on the Recon/Avator-s-10 workstation, including generating fused PET/CT images, delineating regions of interest (ROIs), and measuring standardized uptake values (SUVmax). Scans for the unilateral implantation model were done on day 7 post-implantation, while scans for the bilateral model occurred on days 1, 7, 14, and 21 to observe the uptake and changes of ^18^F-NaF dynamically.

### 2.5. Histopathological Verification

After completing PET/CT scans, the experimental animals were euthanized, and samples from the implantation site and surrounding tissues were collected. The samples were fixed in 4% paraformaldehyde, prepared into paraffin sections, and subjected to HE staining, Masson staining, and toluidine blue staining to examine tissue structure and cell morphology. For the histopathological analysis four consecutive paraffin sections were obtained from the same femoral head of a single rat, representing different anatomical levels of the same site. These sections were used to demonstrate the histological diversity within the femoral head. All slices were derived from one animal (n = 1). Other histological and autoradiographic samples throughout the study were collected from the anatomical locations corresponding to the areas of high radiotracer uptake of the implants in PET/CT imaging in the same rat, ensuring a direct correlation between in vivo imaging and ex vivo analyses. 

### 2.6. Autoradiography

The deparaffinized tissue sections were uniformly coated with a solution containing ^18^F-NaF, incubated for one hour, then washed and dried. The sections were exposed to a phosphor screen, and scanning was performed using the Cyclone Plus phosphor imaging system (PerkinElmer). Distribution of the tracer was analyzed with image analysis software OptiQuant v5 and compared with the results of the pathological staining.

### 2.7. Data Analysis

Statistical methods included the Shapiro–Wilk test for normality assessment, two-way ANOVA to evaluate interaction effects between different age groups and time points, and Pearson or Spearman correlation coefficients for correlation analysis. A significance level of *p* < 0.05 was considered statistically significant, using two-sided tests. Statistical analyses were conducted with SPSS 22.0 and GraphPad Prism 9.0 software.

## 3. Results

### 3.1. Time-Dependent Biodistribution of ^18^F-NaF and ^99m^Tc-MDP in Rat Tissues

**Figure 1:**
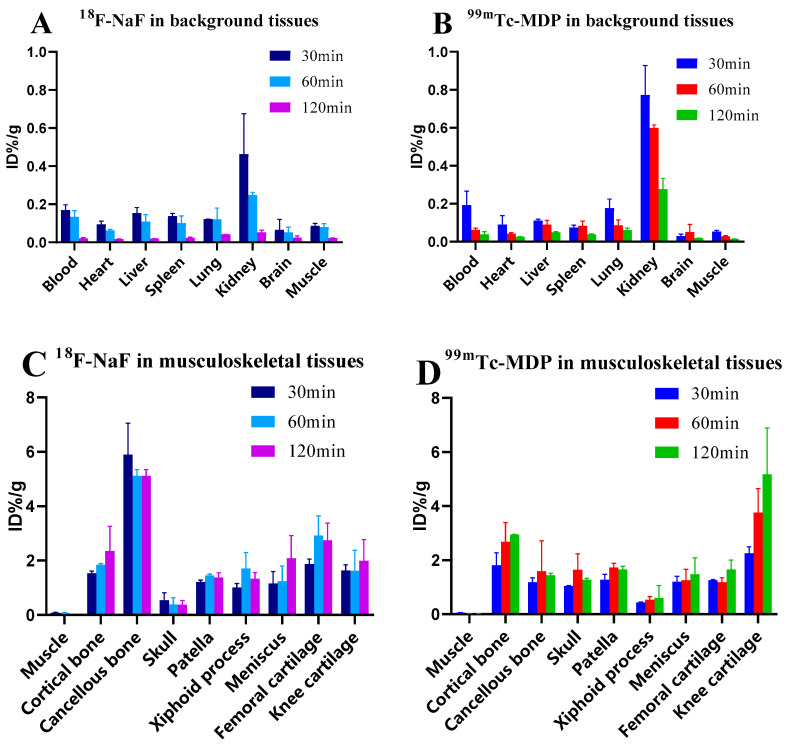
Time-dependent biodistribution of ^18^F-NaF and ^99m^Tc-MDP in different tissues. (**A**) Distribution of ^18^F-NaF in background tissues; (**B**) Distribution of ^99m^Tc-MDP in background tissues; (**C**) Distribution of ^18^F-NaF in musculoskeletal tissues; (**D**) Distribution of ^99m^Tc-MDP in musculoskeletal tissues.

Both ^18^F-NaF and ^99m^Tc-MDP demonstrate declining trends in background tissues over time (Figure 1A,B), with ^18^F-NaF exhibiting faster blood clearance (87.5% reduction from 30–120 min) compared to ^99m^Tc-MDP (79% reduction). In kidneys, liver, spleen, and lungs, ^99m^Tc-MDP maintains significantly higher residual activity at 120 min (0.04–0.28 ID%/g) than ^18^F-NaF (0.02–0.05 ID%/g). In osseous tissues (Figure 1C,D), ^18^F-NaF in cancellous bone shows high initial uptake (5.90 ± 1.15 ID%/g) followed by slight decrease (5.11 ± 0.23 ID%/g), while ^99m^Tc-MDP continuously declines from 1.82 ± 0.16 to 1.45 ± 0.07 ID%/g; cortical bone uptake increases progressively for both tracers—^18^F-NaF by 54% (1.53→2.36 ID%/g) and ^99m^Tc-MDP by 63% (1.81→2.95 ID%/g); skull uptake patterns diverge, with 18F-NaF decreasing (0.54→0.38 ID%/g) and ^99m^Tc-MDP increasing (1.04→1.27 ID%/g).

In cartilaginous tissues, ^18^F-NaF reaches maximum femoral cartilage uptake at 60 min (2.92 ± 0.72 ID%/g) with a subsequent slight decrease (2.75 ± 0.62 ID%/g), maintains relatively stable xiphoid cartilage uptake (1.22→1.33 ID%/g), shows continuous meniscal accumulation (1.16→2.08 ID%/g), and exhibits a modest increase in knee cartilage (1.63→1.99 ID%/g); in contrast, ^99m^Tc-MDP demonstrates remarkable progressive accumulation in knee cartilage (2.26→5.18 ID%/g, 129% increase), becoming the highest uptake site among all tissues, while its femoral cartilage shows a biphasic pattern (1.26→1.18→1.66 ID%/g) and xiphoid cartilage maintains stable low uptake (0.66→0.63 ID%/g), both significantly lower than ^18^F-NaF.

At 120 min, ^18^F-NaF and ^99m^Tc-MDP exhibited significant biodistribution differences (Table 1): ^18^F-NaF demonstrated significantly higher uptake than ^99m^Tc-MDP in cancellous bone (*p* < 0.001), while the opposite pattern was observed in the skull (*p* < 0.001) and patella (*p* = 0.028); regarding cartilaginous tissues, ^18^F-NaF showed significantly greater accumulation in femoral cartilage (*p* = 0.023) and xiphoid cartilage (*p* = 0.010) compared to ^99m^Tc-MDP, whereas ^99m^Tc-MDP exhibited significantly higher uptake in knee cartilage (*p* = 0.015); in background tissues, ^99m^Tc-MDP maintained significantly higher residual activity than ^18^F-NaF in blood (*p* = 0.035), liver (*p* < 0.001), spleen (*p* < 0.001), and lungs (*p* = 0.010), indicating superior background clearance characteristics of ^18^F-NaF.

### 3.2. Age-Related Biodistribution of ^18^F-NaF in Rat Musculoskeletal Tissues

**Figure 2:**
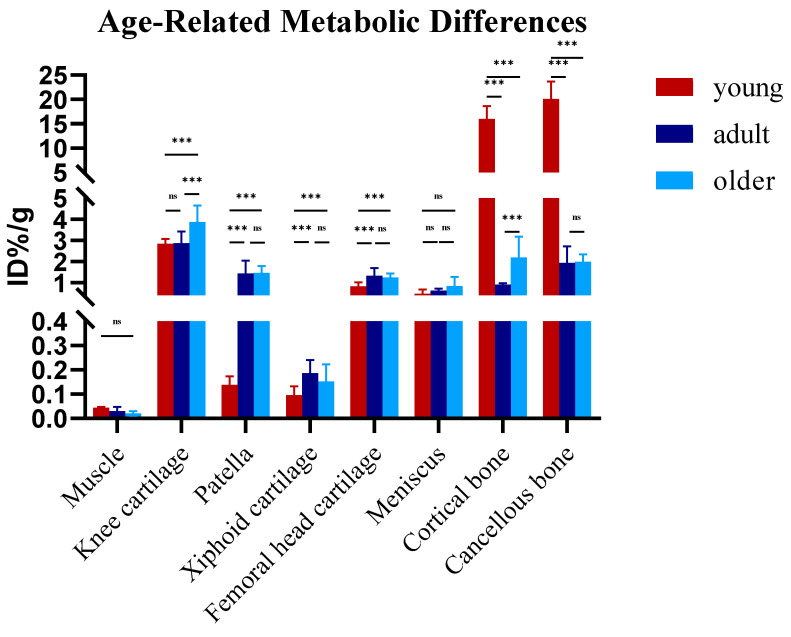
Age-dependent biodistribution of ^18^F-NaF uptake in rat musculoskeletal tissues. Statistical significance is indicated as follows: ***  *p*  <  0.001; ns  *p*  ≥  0.05 (not significant).

The biodistribution of ^18^F-NaF in rat musculoskeletal tissues demonstrates age-specific distribution patterns (Figure 2 and Table 2): cortical bone uptake significantly decreases from juvenile (20.07 ± 3.58) to adult stage (1.94 ± 0.77) and remains stable in aged rats (1.98 ± 0.35) (*p* < 0.001); cancellous bone uptake precipitously declines from juvenile (15.95 ± 2.67) to adult stage (0.91 ± 0.06) followed by significant elevation in aged rats (2.19 ± 0.99) (*p* = 0.009); knee cartilage uptake remains consistent from juvenile (2.84 ± 0.23) to adult stage (2.86 ± 0.56) before significantly increasing in aged rats (3.87 ± 0.79) (*p* = 0.033); femoral cartilage uptake significantly increases from juvenile (0.82 ± 0.19) to adult stage (1.32 ± 0.37) with slight reduction in aged rats (1.25 ± 0.19) (*p* = 0.016); patellar uptake shows marked elevation from juvenile (0.14 ± 0.04) to adult stage (1.44 ± 0.60) and maintains elevated levels in aged rats (1.46 ± 0.32) (*p* < 0.001); meniscus uptake progressively increases from juvenile (0.47 ± 0.20) to adult (0.63 ± 0.09) and aged rats (0.84 ± 0.44) (*p* = 0.075); xiphoid cartilage exhibits an initial increase from juvenile (0.10 ± 0.04) to adult stage (0.19 ± 0.05) followed by a slight decrease in aged rats (0.15 ± 0.07) (*p* = 0.335); skeletal muscle tissue uptake demonstrates a consistent age-dependent decline from juvenile (0.043 ± 0.004) to adult (0.030 ± 0.0167) and aged rats (0.020 ± 0.010) (*p* = 0.055). Juvenile rats display exceptionally high skeletal uptake with relatively low cartilaginous tissue values; adult rats exhibit significantly reduced skeletal uptake with increased weight-bearing cartilage accumulation; aged rats demonstrate maximal knee cartilage uptake, increased cancellous bone accumulation, sustained high levels in other weight-bearing cartilage, and minimal muscle uptake.

### 3.3. Specific Uptake of ^18^F-NaF in Articular Cartilage

**Figure 3:**
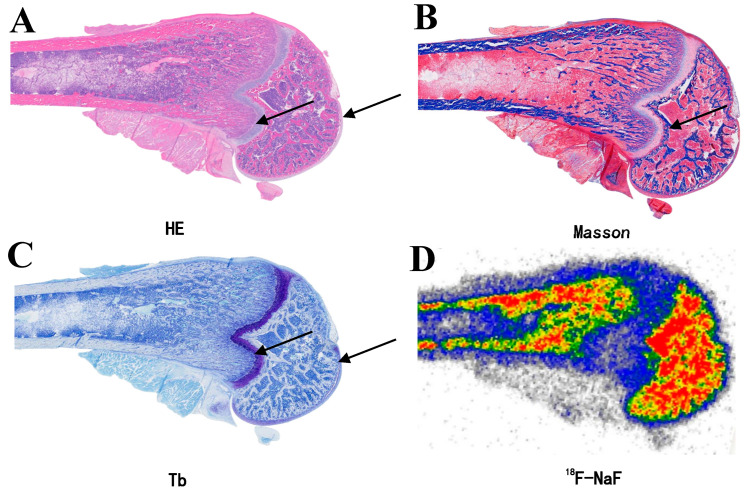
Histopathological staining and autoradiography of the rat femoral head. (**A**) Hematoxylin and Eosin (HE) staining, (**B**) Masson’s trichrome staining, (**C**) toluidine blue staining, and (**D**) ^18^F-NaF autoradiography results. All panels represent consecutive sections from the same rat femur (n = 1).

Quantitative biodistribution analysis revealed a distinct hierarchical uptake pattern (Table 3): cancellous bone showed the highest accumulation (5.11 ± 0.23%ID/g), followed by knee cartilage (1.99 ± 0.78%ID/g), while skeletal muscle exhibited minimal uptake (0.022 ± 0.002%ID/g). The target-to-muscle ratios further emphasized these differences, with the cancellous bone-to-muscle ratio reaching 232.96:1 and the knee cartilage-to-muscle ratio at 90.72:1. Statistical analysis confirmed highly significant differences among the three tissue types (F = 152.63, *p* < 0.0001), with multiple comparison analysis showing significant differences between cancellous bone and knee cartilage, cancellous bone and muscle, and knee cartilage and muscle (all *p* < 0.0001). Histological validation through multimodal imaging techniques provided visual confirmation of these uptake differences. As shown in Figure 3, autoradiography of the rat femoral head (Figure 3D) demonstrated intense ^18^F-NaF accumulation in calcified regions, which corresponded precisely with the calcification front identified by histological staining methods (Figure 3A–C). The spatial distribution of ^18^F-NaF uptake also showed remarkable complementarity with toluidine blue staining (Figure 3C). Importantly, all of these images were obtained from the same rat, ensuring direct comparability among the modalities.

### 3.4. Reasons for Overlooking ^18^F-NaF Uptake Research

**Figure 4:**
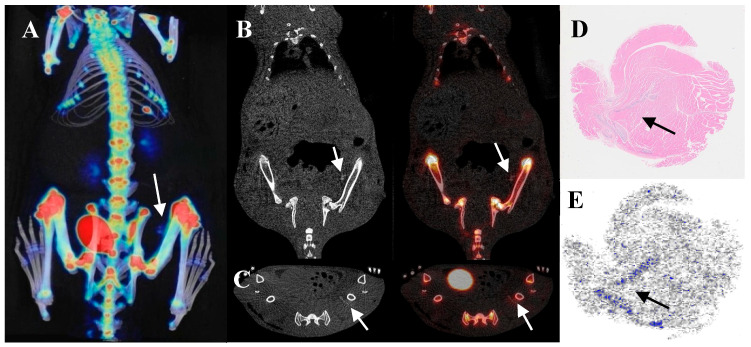
Comparison of ^18^F-NaF uptake between cartilage implantation and sham operation sites in a quadriceps muscle model. The arrows indicate the implant. (**A**) VR fusion image; (**B**) Coronal view; (**C**) Axial view; (**D**) HE staining; (**E**) Autoradiography.

**Figure 5:**
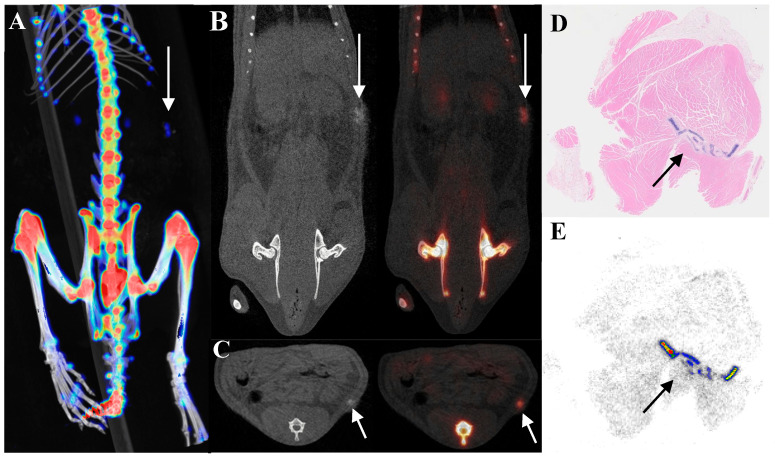
Comparison of ^18^F-NaF uptake between cartilage implantation and sham operation sites in a subcutaneous back model. The arrows indicate the implant. (**A**) VR fusion image; (**B**) Coronal view; (**C**) Axial view; (**D**) HE staining; (**E**) Autoradiography.

Figure 4 illustrates that cartilage uptake is lower than that of bone tissue but higher than that of background tissues. After euthanizing the rats, specimens were collected for HE staining and autoradiography with ^18^F-NaF. HE staining confirmed that the implants were indeed cartilage, while autoradiography revealed radioactive accumulation at the implantation site. This further validated that cartilage can uptake ^18^F-NaF when implanted in vivo. To eliminate potential confounding effects from the proximity of the quadriceps to bone tissue, additional experiments involved subcutaneously implanting cartilage away from the bone in the back. PET/CT imaging for ^18^F-NaF was conducted seven days post-implantation, and results showed radioactive uptake occurring at this distant site, which remained lower than bone tissue but higher than background tissues (Figure 5).

### 3.5. Investigation of Calcification Degree and ^18^F-NaF Uptake

**Figure 6:**
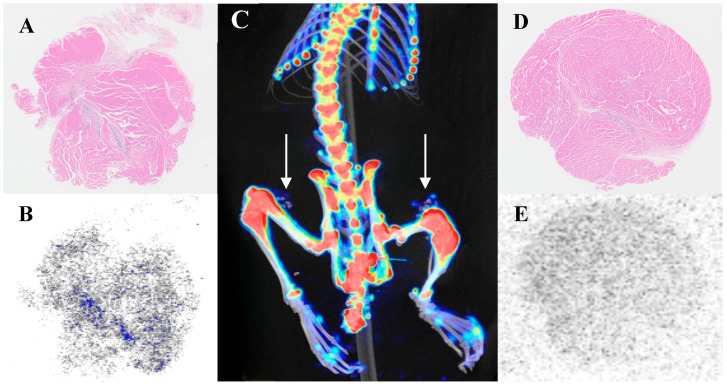
Dependence of ^18^F-NaF uptake on calcification in a bilateral knee cartilage implantation model in quadriceps muscles. The arrows indicate the implant. (**A**) H&E staining of the right-side control implant; (**B**) Autoradiography of the right-side control implant; (**C**) VR fusion image; (**D**) H&E staining of the left-side implant after EDTA decalcification treatment; (**E**) Autoradiography of the decalcified left-side implant.

After implanting cartilage in the bilateral quadriceps, significant uptake of ^18^F-NaF was observed at both implantation sites. Subsequently, both sides were harvested for pathological analysis, with one side undergoing decalcification treatment and the other serving as a normal control. Both tissues were subjected to HE staining and autoradiography analysis. The autoradiography results for the decalcified group showed no uptake, indicating that ^18^F-NaF did not effectively bind or visualize within decalcified tissue. In contrast, the autoradiography of the normal control group displayed expected uptake, supporting the inference that ^18^F-NaF is associated with calcium presence. These results demonstrate that ^18^F-NaF imaging is closely linked to the degree of calcification, and decalcification treatment significantly affects ^18^F-NaF uptake and imaging outcomes, suggesting that ^18^F-NaF imaging primarily reflects the calcification level in tissues (Figure 6).

### 3.6. Dynamic Imaging Changes of Implant Activity

**Figure 7:**
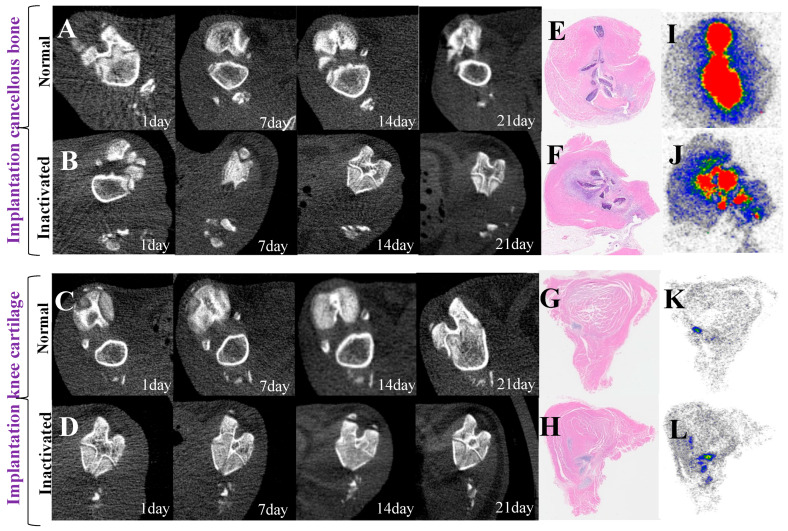
Longitudinal micro-CT imaging and histological analysis comparing normal and inactivated cancellous bone and knee cartilage implants over a 21-day period. (**A**–**D**) Serial micro-CT imaging showing structural and density changes of different implant types: (**A**) Normal cancellous bone implant showing changes from day 1 to day 21. (**B**) Inactivated cancellous bone implant showing changes from day 1 to day 21. (**C**) Normal knee cartilage implant showing changes from day 1 to day 21. (**D**) Inactivated knee cartilage implant showing changes from day 1 to day 21. (**E**–**H**) H&E staining of implants at day 21: (**E**) H&E staining of normal cancellous bone implant. (**F**) H&E staining of inactivated cancellous bone implant. (**G**) H&E staining of normal knee cartilage implant. (**H**) H&E staining of inactivated knee cartilage implant. (**I**–**L**) Autoradiography of corresponding tissues: (**I**) Autoradiography of normal cancellous bone implant. (**J**) Autoradiography of inactivated cancellous bone implant. (K) Autoradiography of normal knee cartilage implant. (**L**) Autoradiography of inactivated knee cartilage implant.

**Figure 8:**
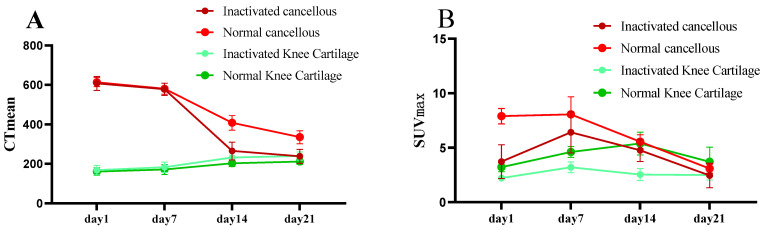
Quantitative analysis of CT values and SUVmax in implanted tissues over time. (**A**) Temporal changes in CT mean values from day 1 to day 21 across four different tissue types. (**B**) Corresponding SUVmax values from 18F-NaF PET imaging of the same tissue types over the identical time period.

Figure 7 and Figure 8 compare the changes in CT values and SUVmax values of implanted cancellous bone and knee cartilage between the inactivated group and the normal group at different time points (1, 7, 14, and 21 days), revealing the impact of cell inactivation treatment on bone and cartilage metabolism (Figure 7A,B). The results showed no significant difference in CT values of cancellous bone between the inactivated and normal groups at early stages (1 and 7 days), indicating that inactivation treatment had a minimal early effect on bone density. However, at 14 and 21 days, the CT values of the inactivated group were significantly lower than those of the normal group (*p* < 0.001), demonstrating that inactivation treatment led to a sustained decrease in bone density and inhibited recovery (Figure 8). The SUVmax at 1 day was significantly lower in the inactivated group compared to the normal group (3.43 ± 1.55 vs. 7.90 ± 0.71, *p* < 0.001), reflecting a significant suppression of metabolic activity in cancellous bone due to inactivation treatment. Although the difference gradually narrowed from 7 to 21 days, the overall metabolic activity continued to show a downward trend (Table 4 and Table 5).

For knee cartilage (Figure 7C,D), CT values showed no significant differences between the inactivated and normal groups at early stages (1 and 7 days), but significantly increased at 14 and 21 days, suggesting that inactivation treatment may have inhibited the recovery of bone density or accelerated calcification in cartilage. The changes in SUVmax exhibited a time-dependent pattern, with the inactivated group significantly lower than the normal group in the early stages, indicating the inhibitory effect of inactivation treatment on cartilage metabolic activity. However, over time, the SUVmax of the inactivated group became significantly higher than that of the normal group at 14 and 21 days, indicating that inactivation treatment enhanced cartilage metabolic activity and calcification processes (Figure 8). This suggests that inactivation treatment accelerated calcification in cartilage, while the metabolic changes in cancellous bone showed an opposite trend, with inactivation treatment leading to a loss of cellular activity and inhibited mineralization (Table 6 and Table 7).

## 4. Discussion

Bone metabolic imaging enables noninvasive evaluation of skeletal metabolism in metastatic disease, fractures, and metabolic disorders [23]. Current clinical tracers include the SPECT agent ^99m^Tc-MDP, which binds hydroxyapatite (HA) via phosphate groups to reflect osteoblastic activity [24,25], and the PET agent ^18^F-NaF, utilizing F^−^/OH^−^ ion exchange within HA to track dynamic calcium deposition [26,27,28,29].While ^18^F-NaF demonstrates superior spatial resolution and target-to-background contrast over ^99m^Tc-MDP, its mechanistic specificity in non-osseous tissues (e.g., weight-bearing cartilage) and regulatory interplay with biomechanical loading remain poorly defined, limiting applications in early osteoarthritis and bone repair monitoring.

This study validated the advantages of ^18^F-NaF imaging by comparing histological uptake differences between ^18^F-NaF and ^99m^Tc-MDP at various time points. These differences may arise from the uptake mechanisms and molecular weight of the two agents: ^18^F-NaF, with its smaller molecular weight and ionic compatibility of fluoride ions (F^−^) with the hydroxyapatite lattice, achieves high specificity in trabecular bone through rapid ion exchange. In contrast, the larger molecular weight of ^99m^Tc-MDP relies on phagocytosis by the reticuloendothelial system, leading to delayed accumulation in cortical bone [30]. Clinical data confirm that the sensitivity and specificity of ^18^F-NaF imaging are significantly higher than those of traditional SPECT, with its rapid blood clearance and improved target-to-background ratio enhancing image quality while reducing radiation dose [31,32,33].

To our knowledge, this is the first study to demonstrate the high specificity of ^18^F-NaF uptake in weight-bearing cartilage, with significant differences in uptake observed among cartilage, bone, and surrounding tissues. Radioautography confirmed that the uptake is localized to the calcification front within the cartilage region. This finding provides a new perspective for the dynamic monitoring of cartilage calcification and may serve as a sensitive imaging biomarker for early osteoarthritis and joint degeneration.

Bone development includes both intramembranous and endochondral ossification, with the latter serving as the primary mechanism for the formation and repair of long bones [4,34]. This process provides the extracellular matrix and mechanical stability through the proliferation, hypertrophy, and mineralization of chondrocytes, facilitating the transition of cartilage to bone tissue. The activities of chondrocytes, calcium salt deposition, and angiogenesis collectively promote this transformation, which is also a critical aspect of fracture repair and bone pathologies [35,36]. The specific uptake mechanism of cartilage for ^18^F-NaF may be related to factors such as calcium salt deposition, cellular activity, mechanical load, and the vascular microenvironment.

Decalcification experiments indicate that the uptake of ^18^F-NaF is entirely dependent on the presence of hydroxyapatite (HA), achieved through ion exchange between fluoride ions (F^−^) and hydroxyl ions (OH^−^) within the HA lattice. After removing HA through decalcification, tissues no longer exhibit uptake of ^18^F-NaF, thereby confirming the specificity of this binding mechanism. Furthermore, previous studies have identified small HA crystal nuclei within cartilage matrix vesicles, which participate in localized Ca^2+^ deposition and serve as critical initiation sites for calcification, widely distributed at the mineralization front in growth plates and at the osteochondral junction [37,38,39,40,41,42]. Notably, the spatial distribution of radioautography exhibits a high degree of complementarity with toluidine blue staining (Figure 3). This complementary feature suggests that the uptake of ^18^F-NaF in cartilage may also be regulated by a locally alkaline microenvironment formed by the degradation of chondroitin sulfate. The local alkaline environment may enhance the binding capacity of F^−^ to HA by promoting calcium salt deposition, thereby synergistically increasing the efficiency of uptake [43,44]. This finding provides new insights into the role of ^18^F-NaF in bone and cartilage metabolism.

Cell viability critically influences ^18^F-NaF uptake in cartilage. The inactivated group exhibited a 60% reduction in ^18^F-NaF uptake compared to healthy controls (*p* < 0.001), confirming the essential role of cellular activity in tracer retention. This phenomenon can be attributed to the regulatory role of chondrocytes in calcium salt deposition, particularly through alkaline phosphatase (ALP) activity. ALP, a key mineralization enzyme, facilitates HA formation by hydrolyzing inorganic pyrophosphate (PPi) to release phosphate ions (Pi) [45,46,47]. Cellular inactivation disrupts ALP functionality, thereby suppressing local calcium deposition and subsequent ^18^F-NaF binding.

Age and mechanical loading jointly modulate bone and cartilage metabolism through skeletal remodeling. Following Wolff’s law, bone tissue dynamically remodels its mineralization patterns in response to mechanical stress magnitude and direction [48]. Younger individuals demonstrate heightened cellular responsiveness to mechanical stimuli, maintaining tissue homeostasis, whereas aging diminishes metabolic activity, leading to matrix degradation, pathological cartilage calcification, and osseous degeneration. Elevated calcium content in weight-bearing cartilage of aged subjects reflects accelerated calcification during degenerative processes [49,50]. Our study demonstrated significantly higher ^18^F-NaF uptake in weight-bearing cartilage (particularly knee cartilage) of aged versus adult groups, with no changes in non-weight-bearing regions, aligning with calcification patterns and emphasizing mechanical load’s metabolic influence.

Notably, inactivated tissues exhibited divergent mineralization trajectories: cartilage displayed progressive increases in density and ^18^F-NaF uptake, while bone showed declining values. This inverse relationship suggests cell death alters mineralization regulatory mechanisms, potentially explaining the co-occurrence of cartilage calcification and osteoporosis in osteoarthritis [51].

^18^F-NaF is widely used for evaluating vascular calcification due to its ability to detect small, active calcified regions in the vascular wall [52,53]. There is notable pathological similarity between cartilage and vascular calcification, particularly regarding their relationship with mechanical stress [54,55,56]. Cartilage calcification occurs in weight-bearing areas, where mechanical stress enhances chondrocyte metabolism and extracellular matrix degradation, thus promoting calcium salt deposition. In contrast, vascular calcification typically arises in regions of high blood flow shear stress, where vascular smooth muscle cells differentiate into osteoblast-like cells, exacerbating the calcification process [57].

In patients with kidney disease, disturbances in calcium and phosphorus metabolism serve as a common pathological basis for vascular calcification, cartilage calcification, and osteoporosis [58]. These types of calcification are interrelated and contribute to the “calcification paradox,” where decreased bone density coincides with increased vascular calcification [59]. This is particularly evident in osteoarthritis patients, who often exhibit both vascular calcification and osteoporosis, highlighting the role of calcium and phosphorus metabolism disorders in systemic calcification pathology [60,61].

^18^F-NaF may be a powerful tool for investigating the shared mechanisms of cartilage and vascular calcification and bone metabolism. Future research integrating molecular imaging, pathology, and biochemical methods could clarify the molecular mechanisms of calcification and its variations across tissues, providing new insights for diagnosing and treating diseases related to systemic calcium and phosphorus metabolism disorders.

Currently, there is a lack of research on the uptake of ^18^F-NaF by cartilage, primarily due to the limitations of traditional bone imaging techniques and a shift in research focus. Cartilage’s thinness and proximity to bone tissue allow the high uptake of ^18^F-NaF by bone to obscure signals from cartilage, especially in lower-resolution SPECT imaging. Moreover, nuclear medicine has concentrated on monitoring bone tissue’s metabolic activities while often overlooking cartilage as mere background tissue. Previous studies have predominantly used maximum standardized uptake value (SUVmax) as the analysis metric, but this is influenced by high-uptake tissues, which can mask true cartilage uptake levels. Additionally, weak signals from cartilage are frequently classified as background noise, resulting in inadequate analysis of its uptake and contributing to its neglect in ^18^F-NaF imaging research [62,63]. Recent advancements in multimodal imaging technologies like PET/MR have enhanced imaging resolution, potentially allowing for differentiation between cartilage and bone metabolic activities. This could lead to new research directions and applications for monitoring cartilage calcification and early diagnosis of osteoarthritis.

This study aims to explore and expand the potential application value of ^18^F-NaF in monitoring metabolic activities of bone and cartilage. Through in vivo and in vitro animal experiments, we compared the uptake differences of ^18^F-NaF with the traditional imaging agent ^99m^Tc-MDP in cartilage and bone tissues. Our results reveal that the uptake of ^18^F-NaF in cartilage is closely related to cellular activity, local calcification, and mechanical loading. Notably, ^18^F-NaF uptake is highly dependent on the presence of hydroxyapatite; in normal adult cartilage, the hydroxyapatite content is extremely low, resulting in minimal ^18^F-NaF accumulation under physiological conditions. This characteristic suggests that ^18^F-NaF imaging may be of limited value for detecting abnormalities in intact cartilage but could serve as a useful tool for excluding cartilage pathology by confirming the absence of uptake. Despite these promising findings, several important limitations should be acknowledged. Foremost, the current study is based on a limited number of animal subjects and, in some histological analyses, single biological replicates (n = 1), which restricts the statistical power and generalizability of the results. Furthermore, as the experiments were conducted exclusively in rat models, substantial biological differences exist between animals and humans, especially regarding cartilage structure and bone metabolism. Therefore, caution should be exercised when extrapolating these preliminary results to the human context. Future studies should clarify the cellular and molecular mechanisms of ^18^F-NaF uptake in cartilage and validate its diagnostic value in human cartilage diseases through larger clinical cohorts. Integrating multimodal imaging techniques will further optimize the dynamic assessment of bone and cartilage metabolism. These efforts will help promote the clinical application of ^18^F-NaF imaging for early diagnosis and management of bone and cartilage disorders.

## 5. Conclusions

In conclusion, this study compared the uptake characteristics of ^18^F-NaF and ^99m^Tc-MDP in various rat tissues through in vivo and in vitro experiments. The results demonstrated that ^18^F-NaF exhibited faster background clearance and a higher target-to-background ratio, particularly in weight-bearing cartilage, where its uptake was closely associated with cellular activity, degree of calcification, and mechanical loading. These findings suggest the potential value of ^18^F-NaF for cartilage metabolic imaging. However, it should be emphasized that the data from this study are still preliminary and cannot yet be widely generalized. Further cellular experiments and clinical studies are needed to validate the clinical application of ^18^F-NaF imaging in bone and cartilage diseases.

## Figures and Tables

**Table 1:** Comparative analysis of the biodistribution of ^18^F-NaF and ^99m^Tc-MDP in different rat tissue types at 120 min.

Tissue Classification	^18^F-NaF	^99m^Tc-MDP	t-Value	*p*-Value
**Bone Tissue**				
femoral condyle (Cancellous)	5.11 ± 0.23	1.45 ± 0.07	31.48	<0.001
femoral cortex (Cortical)	2.36 ± 0.90	2.95 ± 0.01	−1.31	0.24
skull (Flat bone)	0.38 ± 0.16	1.27 ± 0.06	−10.93	<0.001
patella (Sesamoid bone)	1.38 ± 0.18	1.65 ± 0.12	−2.83	0.028
**Cartilage Tissue**				
Femoral cartilage (weight-bearing)	2.75 ± 0.62	1.66 ± 0.35	2.93	0.023
Knee cartilage (weight-bearing)	1.99 ± 0.78	5.18 ± 1.71	−3.39	<0.001
Meniscus (load-bearing area)	2.08 ± 0.83	1.48 ± 0.61	1.34	0.230
Xiphoid process (hyaline cartilage)	1.33 ± 0.23	0.60 ± 0.46	3.71	0.010
**Background Tissue**				
Blood (blood pool)	0.020 ± 0.005	0.039 ± 0.014	−2.70	0.035
Liver (metabolism)	0.019 ± 0.001	0.049 ± 0.004	−15.81	<0.001
Spleen (reticuloendothelial)	0.023 ± 0.005	0.039 ± 0.002	−6.32	<0.001
Lung (capillaries)	0.042 ± 0.001	0.061 ± 0.010	−3.71	0.010

**Table 2:** Comparative analysis of age-dependent biodistribution parameters in various rat tissues. Statistical significance is indicated as follows: **  *p*  <  0.01; ***  *p*  <  0.001; **** *p* < 0.0001.

Tissue Classification	Juvenile (4 Weeks)	Adult (4 Months)	Aged (14 Months)	F-Value	*p*-Value
Cortical bone	20.07 ± 3.58 ****	1.94 ± 0.77	1.98 ± 0.35	152.73	<0.001
Cancellous bone	15.95 ± 2.67 ***	0.91 ± 0.06	2.19 ± 0.99 **	98.56	0.009
Knee cartilage	2.84 ± 0.23	2.86 ± 0.56	3.87 ± 0.79 ***	4.28	0.033
Femoral cartilage	0.82 ± 0.19	1.32 ± 0.37 **	1.25 ± 0.19 **	5.92	0.016
Meniscus	0.47 ± 0.20	0.63 ± 0.09	0.84 ± 0.44	3.1	0.075
Patella	0.14 ± 0.04	1.44 ± 0.60 **	1.46 ± 0.32 **	21.5	<0.001
Xiphoid cartilage	0.10 ± 0.04	0.19 ± 0.05	0.15 ± 0.07	1.2	0.335
Muscle	0.043 ± 0.004	0.030 ± 0.0167	0.020 ± 0.010	4.1	0.055

**Table 3:** Comparison of target uptake ratios in musculoskeletal tissues and multiple comparison analysis.

Tissue Type	Mean ± SD (%ID/g)	Target-to- Muscle Ratio	F-Value	*p*-Value	Post Hoc Comparisons
Cancellous Bone (Bone Tissue)	5.11 ± 0.23	232.96:1	152.63	< 0.0001	Cancellous Bone vs. Knee Cartilage: *p* < 0.0001
Knee Cartilage (Cartilage Tissue)	1.99 ± 0.78	90.72:1			Cancellous Bone vs. Muscle: *p* < 0.0001
Quadriceps Muscle (Background Tissue)	0.022 ± 0.002				Knee Cartilage vs. Muscle: *p* < 0.0001

**Table 4:** CT values of implanted cancellous bone at different time points.

Time	Group Comparison	Mean ± SD (CT Mean)	t-Value	*p*-Value
1 day	Inactivated vs. Normal	607.34 ± 35.55 vs. 613.97 ± 22.85	−0.31	0.762
7 days	Inactivated vs. Normal	578.15 ± 30.92 vs. 580.67 ± 27.38	−0.15	0.886
14 days	Inactivated vs. Normal	265.45 ± 45.11 vs. 408.01 ± 37.23	−5.12	<0.001
21 days	Inactivated vs. Normal	238.26 ± 35.62 vs. 325.87 ± 32.71	−4.89	<0.001

**Table 5:** SUVmax values of implanted cancellous bone at different time points.

Time	Group Comparison	Mean ± SD (SUVmax)	t-Value	*p*-Value
1 day	Inactivated vs. Normal	3.43 ± 1.55 vs. 7.90 ± 0.71	−5.72	<0.001
7 days	Inactivated vs. Normal	6.34 ± 1.89 vs. 8.07 ± 1.61	−1.84	0.107
14 days	Inactivated vs. Normal	4.77 ± 1.03 vs. 5.56 ± 0.65	−1.65	0.142
21 days	Inactivated vs. Normal	2.46 ± 1.13 vs. 3.09 ± 0.42	−1.34	0.225

**Table 6:** CT values of implanted knee cartilage at different time points.

Time	Group Comparison	Mean ± SD (CT Mean)	t-Value	*p*-Value
1day	Inactivated vs. Normal	167.82 ± 24.19 vs. 160.86 ± 19.37	0.54	0.606
7days	Inactivated vs. Normal	182.67 ± 26.13 vs. 171.78 ± 25.12	0.79	0.457
14days	Inactivated vs. Normal	232.41 ± 14.74 vs. 202.82 ± 16.38	3.41	0.014
21days	Inactivated vs. Normal	239.34 ± 21.15 vs. 211.49 ± 13.80	2.64	0.032

**Table 7:** SUVmax values of implanted knee cartilage at different time points.

Time	Group Comparison	Mean ± SD (SUVmax)	t-Value	*p*-Value
1 day	Inactivated vs. Normal	2.22 ± 0.49 vs. 3.22 ± 0.41	−3.52	0.008
7 days	Inactivated vs. Normal	3.21 ± 0.49 vs. 4.62 ± 0.50	−4.89	0.001
14 days	Inactivated vs. Normal	2.55 ± 0.55 vs. 5.39 ± 1.04	−5.67	<0.001
21 days	Inactivated vs. Normal	2.49 ± 0.44 vs. 3.72 ± 1.34	−2.56	0.032

## Data Availability

The datasets generated during and/or analyzed during the current study are available from the corresponding author on reasonable request.

## Abbreviations

^18^F-NaF	^18^F-Sodium Fluoride
^99m^Tc-MDP	^99m^Tc-Methylene Diphosphonate
PET	Positron emission tomography
HA	Hydroxyapatite
ALP	Alkaline phosphatase
PPi	Pyrophosphate
Pi	Phosphate ions
